# Taxonomic reassessment of fossil *Sequoia* and *Protosequoia* from the Upper Miocene of Central Honshu, Japan, with implications for leaf morphological variation in extant *S. sempervirens*

**DOI:** 10.1002/ajb2.70227

**Published:** 2026-06-24

**Authors:** Shun Ikeda, Arata Momohara

**Affiliations:** ^1^ Graduate School of Horticulture Chiba University 648 Matsudo Chiba 271‐8510 Japan

**Keywords:** Cupressaceae, Japan, Late Miocene, leaf morphological variation, plant macrofossils, *Protosequoia*, seasonal drought, *Sequoia*, taxonomic revision

## Abstract

**Premise:**

Since its emergence in the Mesozoic, *Sequoia* (Cupressaceae) has been considered to possess conserved leaf morphology. However, recent studies have shown that the leaves of extant *S. sempervirens* become smaller, with a scale form, with increasing tree height. The fossil‐genus *Protosequoia* has a similar scale leaf morphology, but its relationship to co‐occurring *Sequoia* fossils, which bear linear leaves, remains unresolved.

**Methods:**

To resolve this relationship, we re‐examined specimens of two fossil species of *Sequoia* and *Protosequoia* from the Upper Miocene of Japan, focusing on leaf epidermal morphology and vascular bundle structure in the bract–scale complexes of seed cones.

**Results:**

We identified fossil specimens with two types of shoots that were preserved in organic connection, but they had previously been classified as different genera. As the leaf shape changed from linear to scale, stomatal orientation shifted from parallel to the midrib to irregular, and epidermal cells gradually became shorter. Seed cones contained fewer than 30 bract–scale complexes with rhombic distal surfaces marked by horizontal grooves. In cross section, scales had rhombically arranged vascular bundles and several abaxial resin canals. Seeds had broad wings, and male strobili were mostly spherical.

**Conclusions:**

The connected shoots, which shift in leaf stomatal features, and similarities in reproductive structure of the fossils suggest that the two genera represent the same species. Therefore, we propose a new *Sequoia* species characterized by densely branched twigs bearing smaller scale leaves, possibly reflecting an adaptation to different climatic conditions, compared to other species.

*Sequoia sempervirens* (D. Don) Endl. (Cupressaceae) is endemic to the Pacific coast of North America and is the tallest tree species in the world, reaching heights exceeding 115 m. Although the genus *Sequoia* is represented today by only one living species, it was more diverse in the past. Before the Pliocene, multiple fossil species of *Sequoia* were distributed across the northern hemisphere (Zhang et al., 2015), and fossil genera considered closely related to *Sequoia*, including *Quasisequoia* (Kunzmann, 1999), have also been described. In the Cretaceous, fossils that have been assigned to *Sequoia* and *Sequoia*‐related genera became even more numerous; many of these were as assigned to separate genera primarily on the basis of differences in the anatomical features of seed cones (Ohsawa et al., 1992; Ohsawa, 1994; Rothwell and Ohana, 2016; Sokolova et al., 2017).

Nonetheless, *Sequoia* has long been considered to have maintained relatively unchanged leaf morphology since the Mesozoic era, based on fossils that closely resemble extant *S. sempervirens* (Zhang et al., 2015; Ma et al., 2021). However, recent studies of extant *S. sempervirens* have revealed substantial variations in leaf morphology within a single tree, depending on physiological conditions, which vary with height (Koch et al., 2004; Ishii et al., 2008; Oldham et al., 2010; Ishii et al., 2014; Van Pelt et al., 2016; Chin et al., 2022). In tall trees, lower shoots predominantly bear linear leaves, and leaf size gradually decreases with increasing height. In the upper canopy, at heights over approximately 80 m, shoots bear only short, thick, scale‐like leaves (Koch et al., 2004; Van Pelt et al., 2016). This evidence suggests that some fossil species previously classified as distinct based on differences in leaf morphology may actually represent morphological variations within a single tree.

In Japan, a fossil taxon characterized by shoots bearing only scale leaves, *Protosequoia primaria* (Miki) Miki, was described as a distinct fossil genus separate from *Sequoia* (Miki, 1969). This species has been documented in the Upper Miocene Seto and Tokiguchi Porcelain Clay Formations and co‐occurs with fossil *Sequoia* species that bear linear leaves (Miki, 1941, 1965). Therefore, to determine whether *Protosequoia* is taxonomically distinct, it is essential to investigate the morphological transition from scale leaves to linear leaves in modern *Sequoia* trees.

In most descriptions, fossil *Sequoia* species are recorded as bearing linear leaves (Kvaček, 1976; Zhang et al., 2015). This oversight in leaf shape variation may have led to the misidentification of the same species as different taxa, potentially influencing discussions on their evolution.

In this study, we quantitatively assessed the variation in leaf epidermal morphology within shoots bearing *Sequoia*‐type linear leaves and *Protosequoia*‐type scale leaves from the Miocene Seto Group. We then compared these results with the variation observed in extant *S. sempervirens* and examined modern and fossil reproductive organs, to assess the taxonomic position and paleoecology of *P. primaria*.

## MATERIALS AND METHODS

**Figure 1:**
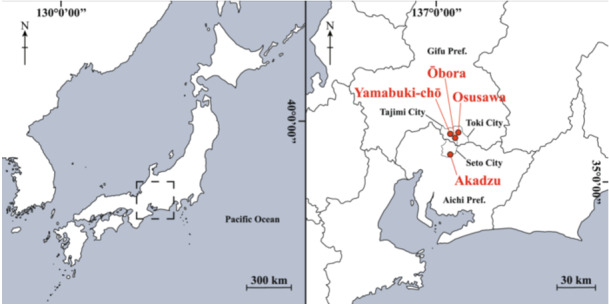
Localities of examined specimens on island of Honshu, Japan. Pref., Prefecture.

Fossil specimens collected by Dr. Shigeru Miki, including *Sequoia* and *Protosequoia* described by Miki (1963, 1965, 1969), are stored at the Osaka City Museum of Natural History, Osaka, Japan (Herbarium Code: OSA) (Kokawa et al., 2006). The specimens used in this study were primarily collected from the Akadzu locality of the Seto Porcelain Clay Formation in Seto City, Aichi Prefecture, Japan (Miki, 1969; Figure 1) and from several localities of the Tokiguchi Porcelain Clay Formation in Tajimi and Toki cities, Gifu Prefecture, Japan (Miki, 1965; Figure 1). The fossil specimens from Akadzu, including *Protosequoia*, as described by Miki (1969), were either mounted on glass slides or stored in glass jars. The type locality of *Protosequoia* was originally reported as the “Kibushi clay mine near the border between Tokitsu and Tajimi” (Miki, 1965). However, we confirmed that the specimen is labeled with the locality name “Ōbora.” Fossils identified as *Sequoia* have been found in a wider range of localities than *Protosequoia*, including Osusawa, located east of Ōbora, where numerous pinnate shoot fossils co‐occur with *Protosequoia* (Miki, 1941; Kokawa et al., 2006). The Seto and Tokiguchi Porcelain Clay Formations, which are distributed across these areas, have been dated to approximately 11–9 million years ago (Ma) based on fission track dating of several tephra layers and are correlated with the late Middle Miocene to the early Late Miocene (Hatano and Yoshida, 2018). For this study, we examined specimens mounted on glass slides from the three localities (Akadzu, Ōbora, and Osusawa) and fossil shoot specimens stored in 70% v/v ethanol in glass jars (Akadzu: OSA F17473; Ōbora: OSA F19405; Osusawa: OSA F20407), which originated from the same localities as the specimens on glass slides. At the time of our study, the label for specimen bottle OSA F17473 had not been inserted. Based on the museum's Excel catalogue entry “Akadzu (estimated)” and a comparison with the morphology described by Miki (1969), the specimen was determined to have originated from Akadzu. We examined these specimens to analyzed their epidermal morphology.

In addition to the fossil specimens collected by Dr. Miki, we analyzed fossil samples obtained from an organic layer (P1) in a sand–silt alternation of the Tokiguchi Porcelain Clay Formation in Yamabuki‐chō, Tajimi City, Gifu Prefecture, Japan (35.34601° N, 137.14703° E; Momohara and Saito, 2001; Figure 1). This locality is near the border between Tajimi and Toki Cities and may correspond to one of the Ōbora localities described by Dr. Miki. A tephra layer dated to 9.7 ± 0.4 Ma using the fission‐track method (Ando et al., 1999) is present below P1, corresponding to the early Late Miocene (Momohara and Saito, 2001). Fossil specimens are stored at the Graduate School of Horticulture, Chiba University, Matsudo, Chiba, Japan (Herbarium Code: MTDO). Samples were soaked in a 1% w/v KOH solution to macerate the sediment and then washed through a sieve to separate plant fossils from inorganic materials. Particularly well‐preserved specimens were sealed in plastic films containing 70% v/v ethanol. The other fragmentary branches were stored in glass jars containing 70% v/v ethanol.

Samples of modern *S. sempervirens* were obtained from individuals planted at the Tsukuba Botanical Garden, National Museum of Nature and Science, Tsukuba, Ibaraki, Japan, and from wild individuals at Prairie Creek Redwoods State Park, Humboldt County, California, USA. The sample from the Tsukuba Botanical Garden was collected in June 2023 from a branch 5 m above the ground on an individual approximately 25 m tall. Shoots bearing sun leaves were collected from branches in the sun‐exposed canopy; shoots with shade leaves were collected from shaded branches near the trunk. Cones were collected from fallen shoots beneath the tree. The California samples were obtained from a tree named Zeus (Ishii et al., 2008), approximately 101 m tall, and were stored at the Faculty of Agriculture, Kobe University, Kobe, Hyōgo Prefecture, Japan. These samples were collected in autumn 2000 from shoots at heights of 25, 55, 70, 85, and 100 m in the outer canopy. All studied specimens are currently stored at the Graduate School of Horticulture, Chiba University.

To examine epidermal morphology, we immersed leaf fossils in Schultze solution (2:1 concentrated HNO_3_ and saturated KClO_3_), on a hot plate at 40°C for 1 h. The fossils were then soaked in a 10% w/v KOH solution for approximately 10 min and rinsed with water. The cuticle layers were stained with a 1% w/v safranin solution in 50% v/v ethanol for 3 min. The samples of extant species were soaked in a mixture of 10% w/v NaOH) and 10% H_2_O_2_ for 24 h, then washed with water, and soaked in a 20% w/v chromium (VI) oxide (CrO_4_) solution on a hot plate at 40°C for 24 h to separate the cuticle layers.

To examine how epidermal morphology changes with differences in leaf length on the same branchlet, we measured stomatal angle and epidermal cell length using branchlets from fossil shoots from different localities and different heights of modern trees. For each branchlet, three leaves—a scale leaf at the base, a linear leaf from the middle of a branchlet, and a leaf of intermediate length from the middle—were measured. Three branchlets were examined from each of the four fossil localities (Yamabuki‐chō: MTDO f‐1002‐13; Akadzu: OSA F17473; Ōbora: OSA F19405; Osusawa: OSA F20407), and five branchlets were examined for each height class of the extant specimens. Branchlets from the 100‐m height class of the extant specimens were excluded because they bore only scale leaves. Epidermal structures were measured at the central part of each leaf using ImageJ version 1.53t (National Institutes of Health, Bethesda, MD, USA).

To examine the anatomical features of seed cone scales, the scales were embedded in Technovit 7100 (Kulzer GmbH, Hanau, Germany) as described by Tobe and Kadokawa (2014) and sectioned using a sliding microtome (Retoratome REM‐710, Yamato Kohki Industrial, Niiza, Japan). Epidermal and sectioned samples were mounted on glass slides using Softmount (FUJIFILM Wako Pure Chemical, Osaka, Japan). Specimens were photographed and measured using a stereomicroscope (SMZ18, Nikon, Tokyo, Japan) equipped with a microscope camera (DS‐Ri2, Nikon). Some epidermal samples were Au‐coated using a Fine Coat ion sputter (JFC‐1100, JEOL Ltd., Akishima, Japan) and imaged with a scanning electron microscope (SU1510, Hitachi High‐Tech, Tokyo, Japan) at 15 kV. Leaves with a twisted base, causing the adaxial side to face upward, were classified as linear leaves; leaves without a twisted base, with the abaxial side facing outward, were classified as scale leaves.

## RESULTS AND SYSTEMATICS

### Morphological continuity between fossil *Sequoia* and *Protosequoia*

**Figure 2:**
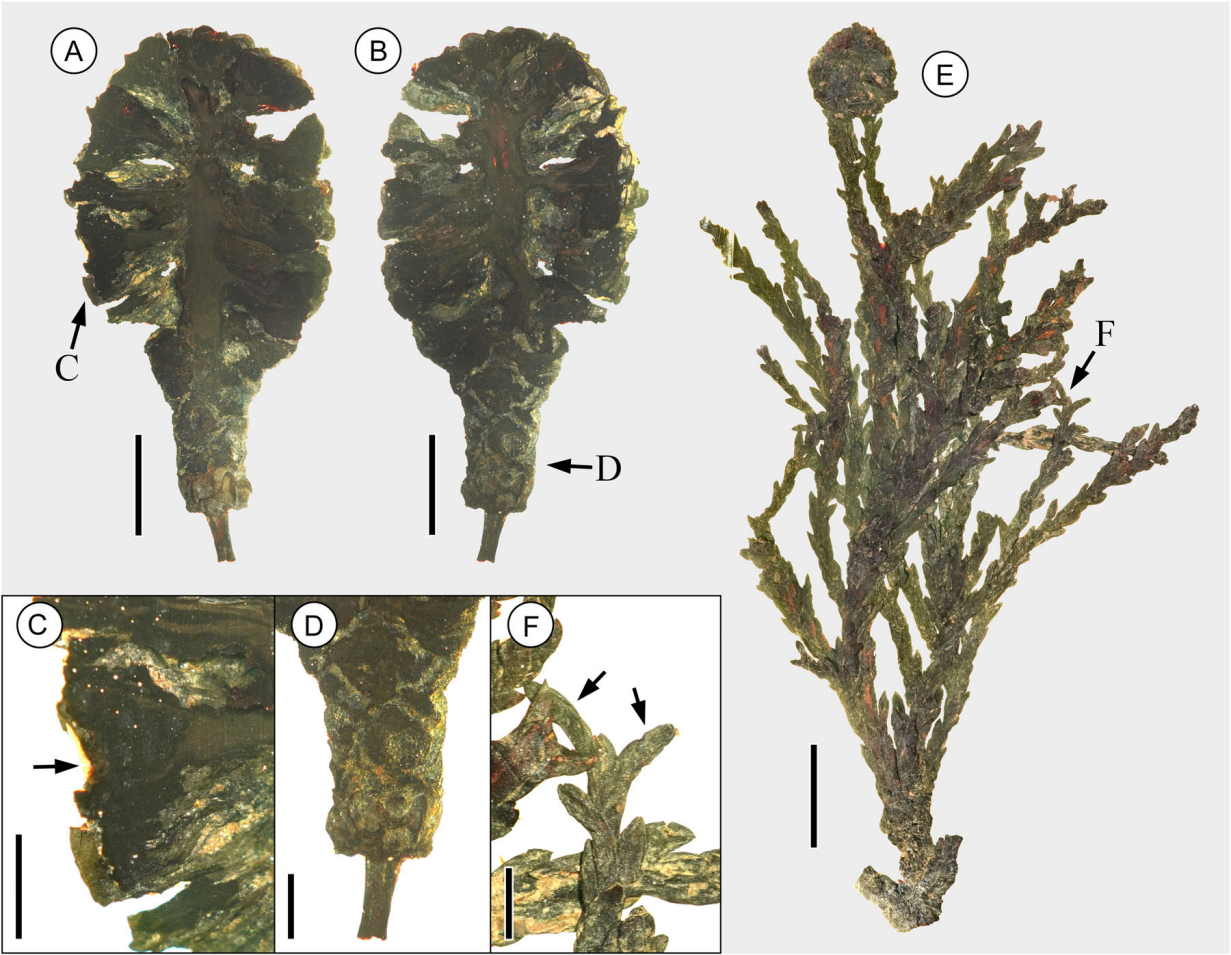
*Sequoia primaria* comb. nov. (A–D) Lectotype specimen (OSA F11829; Miki, 1965, Plate 1Cb; Ōbora locality). (A) Longitudinal section of mature seed cone (arrow: area of close‐up in C); (B) external view of same seed cone, reverse side of (A) (arrow: area of close‐up in D); (C) close‐up of groove (arrow) on distal surface of bract–scale complex; (D) close‐up of densely arranged scale leaves at base of seed cone. (E, F) Epitype specimen (OSA F2070; Miki, 1969, Figure 1 Aa; Akadzu locality). (E) Shoot bearing immature seed cone (arrow: area of close‐up in F); (F) close‐up of small linear leaves (arrows) on right branchlet. Scale bars: A, B = 5 mm; E = 10 mm; C, D, F = 2 mm.

**Figure 3:**
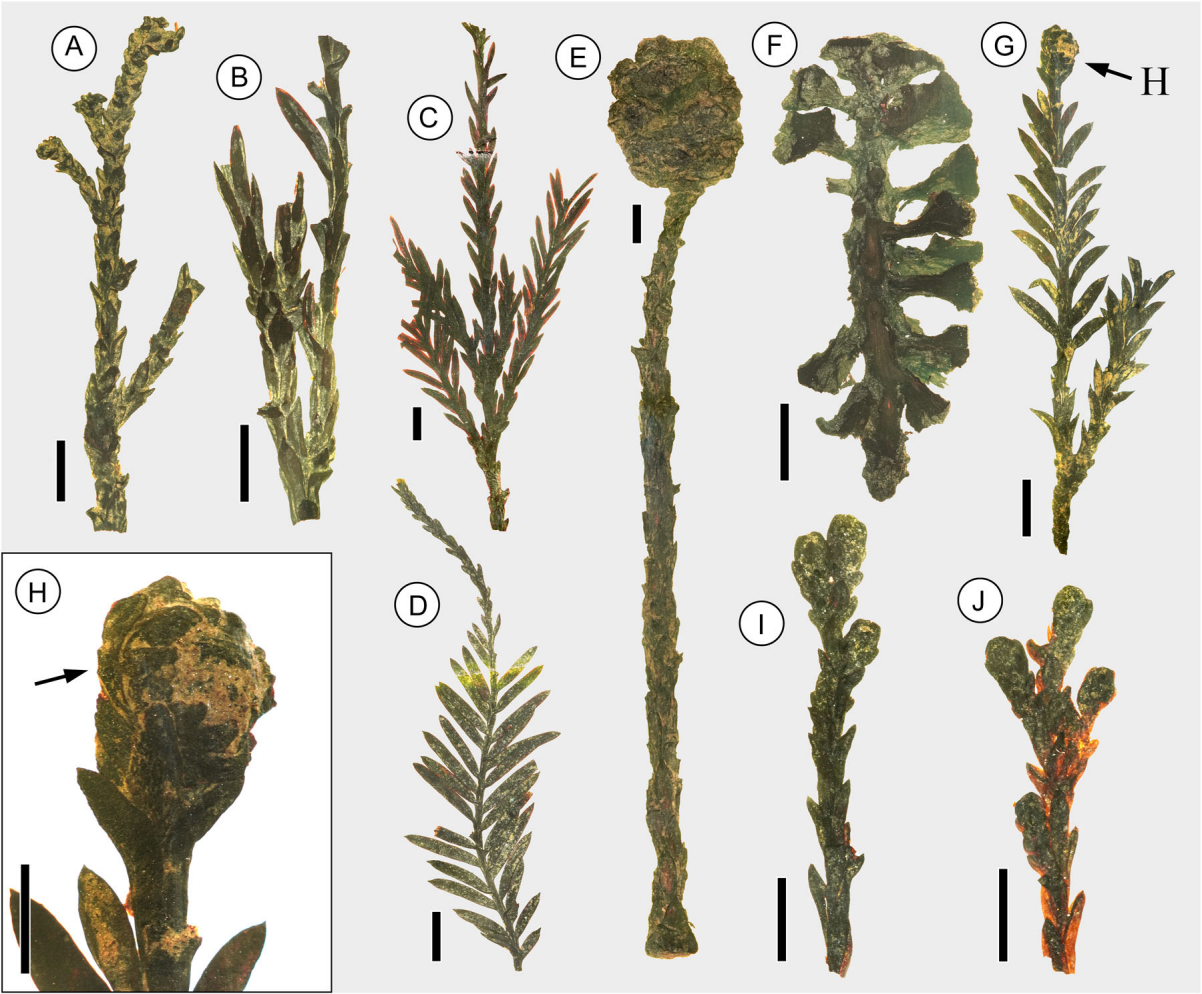
*Sequoia primaria* comb. nov. (A–D) Shoots: (A) OSA F2296 (Miki, 1965, Plate 1Aa; Ōbora locality); (B) OSA F12132 (Ōbora locality); (C) OSA F11527 (Osusawa locality); (D) OSA F2630 (Miki, 1941, Plate 5 F; Osusawa locality). (E) Seed cone attached to a shoot with scale leaves, OSA F2069 (Akadzu locality). (F) Vertical section of seed cone, OSA F2194 (Miki, 1965, Plate 1 Cd; Ōbora locality). (G) Shoot with mature male strobilus, OSA F2618 (Akadzu locality) (arrow: area of close‐up in H). (H) Close‐up of male strobilus showing several microsporophylls (arrow). (I, J) Shoots with immature male strobili, OSA F2657 (Miki, 1965, Plate 1E; Ōbora locality). Scale bars: A–G, I, J = 5 mm; H = 2 mm.

**Figure 4:**
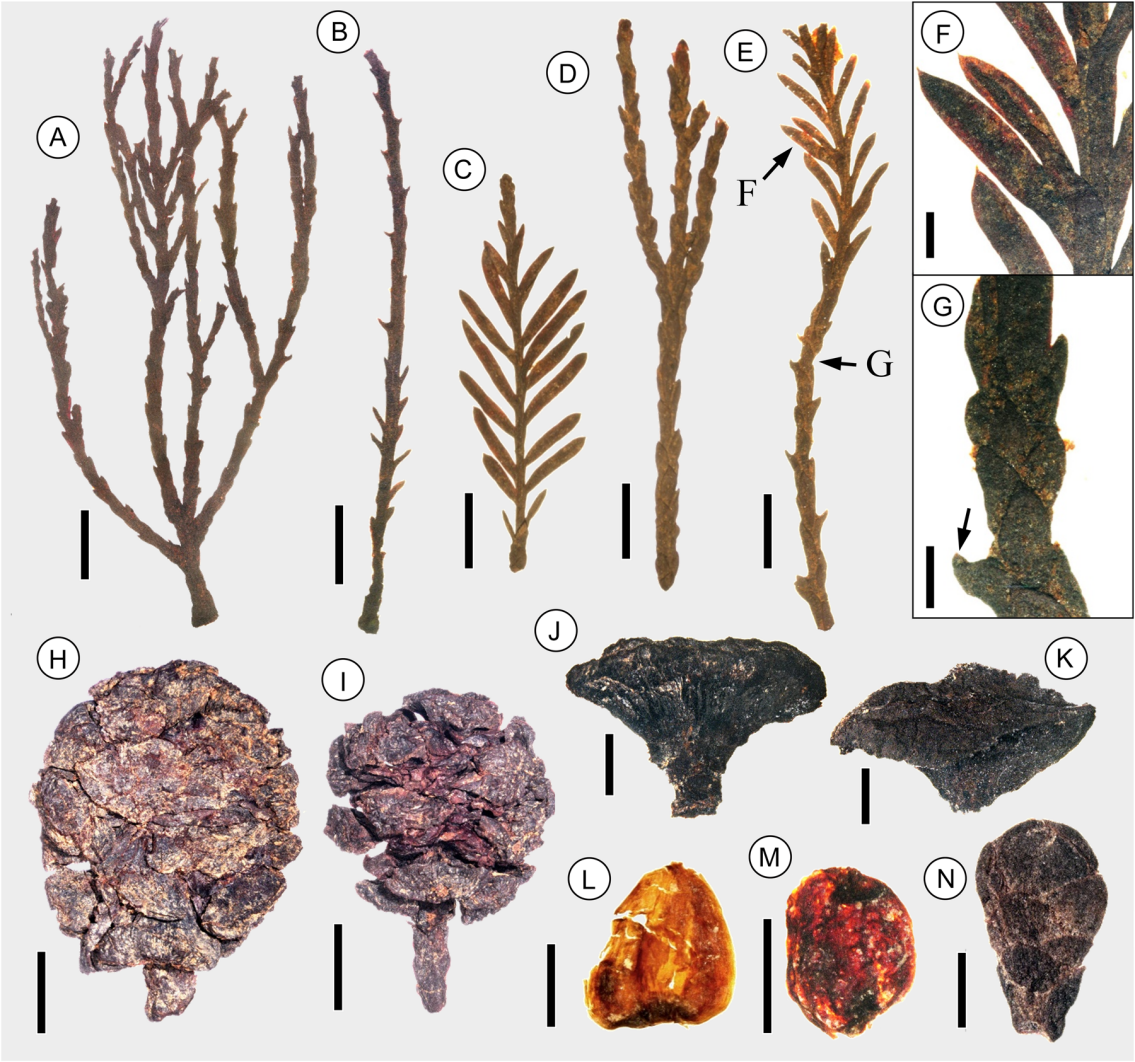
*Sequoia primaria* comb. nov. (Yamabuki‐chō locality). (A–G) Shoots: (A) MTDO f‐1002‐1; (B) MTDO f‐1002‐2; (C) MTDO f‐1002‐3; (D) MTDO f‐1002‐4; (E) MTDO f‐1002‐5 (arrows: area of close‐ups in F and G); (F) Close‐up of linear leaves with a broad base, attached along the shoot; (G) close‐up of scale leaves, densely attached at base of a shoot with linear leaves. Arrow indicates irregularly spreading short subulate leaf within a scale‐leaved shoot. (H, I) Seed cones: (H) MTDO f‐1002‐6; (I) MTDO f‐1002‐7. (J, K) Bract–scale complexes: (J) MTDO f‐1002‐8; (K) MTDO f‐1002‐9. (L, M) Seeds: (L) MTDO f‐1002‐10; (M) MTDO f‐1002‐11. (N) Immature male strobilus, MTDO f‐1002‐12. Scale bars: A, B = 10 mm; C–E, H, I = 5 mm; J–N = 2 mm; F, G = 1 mm.

We observed several morphological similarities and continuity between a fossil shoot primarily composed of scale leaves, identified as *Protosequoia* by Dr. Miki, and a fossil shoot bearing linear leaves, identified as *Sequoia*. The *Protosequoia* fossil shoot specimen presented by Dr. Miki (1969: Figure 1), composed primarily of scale leaves, bore a few short linear leaves (Figure 2F). Similarly, in fossil *Sequoia*, shoots with typical linear leaves had scale leaves at the apex or base and showed a gradual morphological transition between the two leaf types (Figures 3B, 3D, 3G, 4C, 4E). Furthermore, many fossil shoots contained leaves with intermediate forms between scale and linear types on the same shoot (Figures 3B–D, 3G, 4B, 4C, 4E).

**Figure 5:**
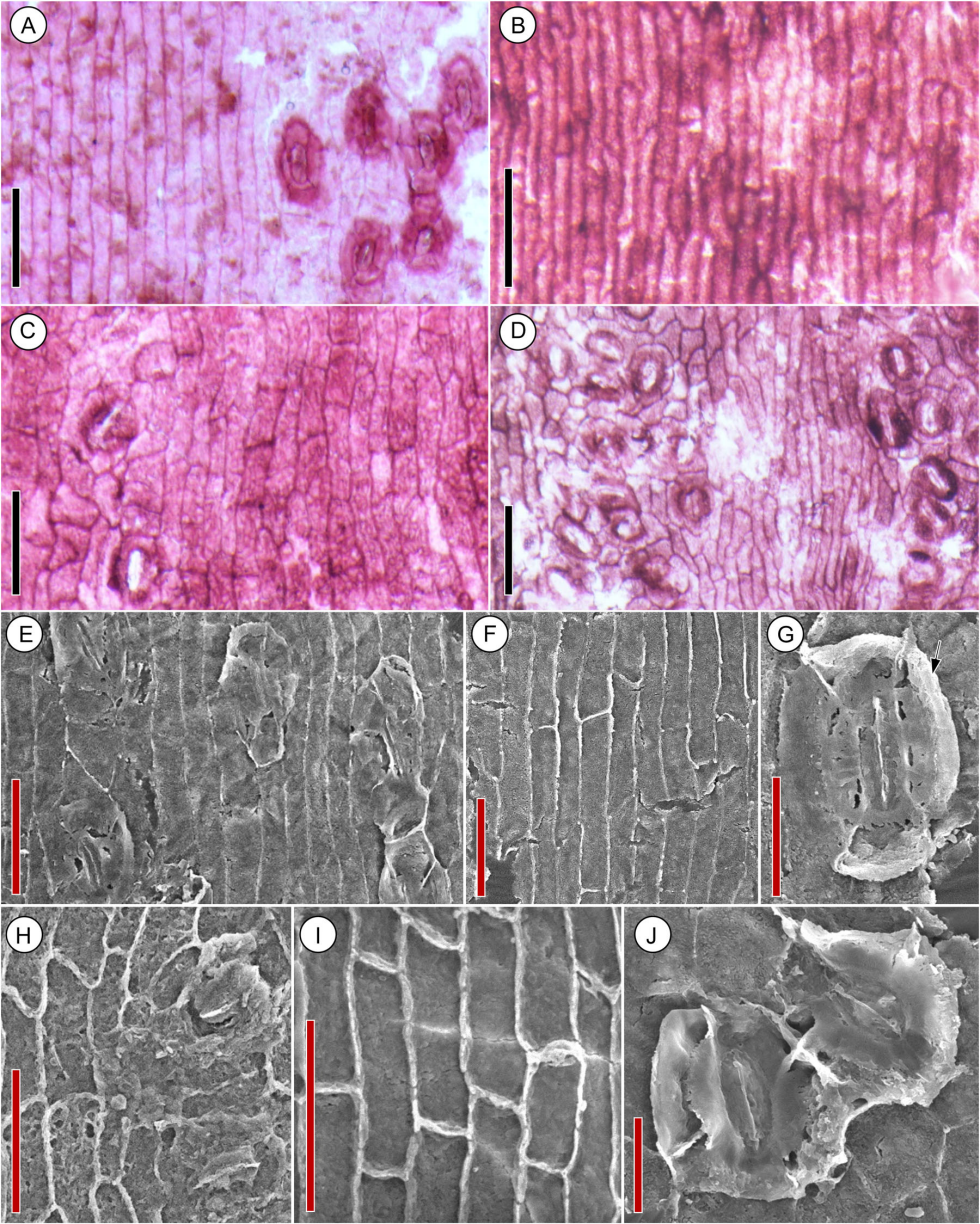
Leaf epidermal structure of *Sequoia primaria* comb. nov. (Yamabuki‐chō locality). (A) Abaxial surface of linear leaf, with numerous stomata arranged parallel to elongated, rectangular epidermal cells (MTDO f‐1002‐17). (B) Adaxial surface of linear leaf, with slightly shorter epidermal cells and very few or no stomata compared to the abaxial surface (MTDO f‐1002‐18). (C) Abaxial surface of scale leaf, with shorter epidermal cells and a few stomata arranged in several irregular rows (MTDO f‐1002‐30). (D) Adaxial surface of scale leaf, showing significantly more stomata than on abaxial surface (MTDO f‐1002‐25). (E) SEM image of abaxial surface of linear leaf, with stomata oriented parallel to midrib (MTDO f‐1002‐34). (F) SEM image of epidermal cells on abaxial surface of linear leaf (MTDO f‐1002‐34). (G) SEM image of stoma on abaxial surface of linear leaf; arrow indicates one of four subsidiary cells (four i (MTDO f‐1002‐34). (H) SEM image of abaxial surface of scale leaf, showing irregularly oriented stomata (MTDO f‐1002‐35). (I) SEM image of epidermal cells on abaxial surface of scale leaf (MTDO f‐1002‐36). (J) SEM image of stomata on abaxial surface of scale leaf (MTDO f‐1002‐36). Scale bars: A–D = 100 µm; E, F, H, I = 50 µm; G, J = 20 µm.

**Figure 6:**
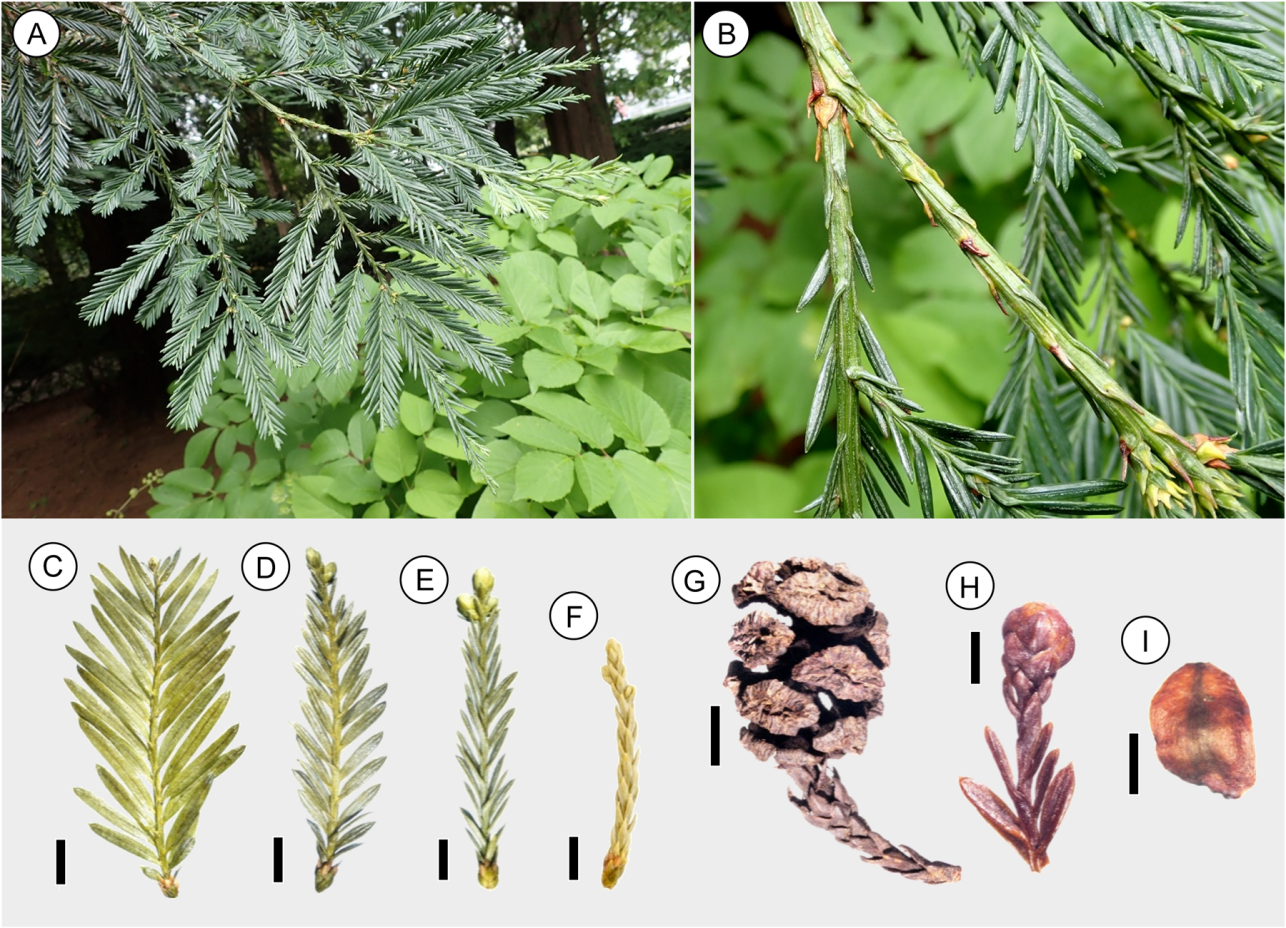
External morphology of extant *Sequoia sempervirens* leaves at different heights and reproductive structures. Samples were obtained from a cultivated tree at the Tsukuba Botanical Garden, Tsukuba, Japan (A, B, G–I) and from the wild tree named Zeus at Prairie Creek Redwoods State Park, California, United States (C–F). (A) Sun‐exposed shoots from approximately 5 m above the ground. (B) Close‐up of central portion of shoots in (A); shoots occasionally develop with scale leaves spaced apart. (C) Branchlet from a height of (C) 25 m, (D) 70 m, (E) 85 m, (F) 100 m. (G) Seed cone. (H) Seed with thick lateral wings. (I) Immature male strobilus at branchlet tip. Scale bars: C–F = 5 mm; G–I = 2 mm.

**Figure 7:**
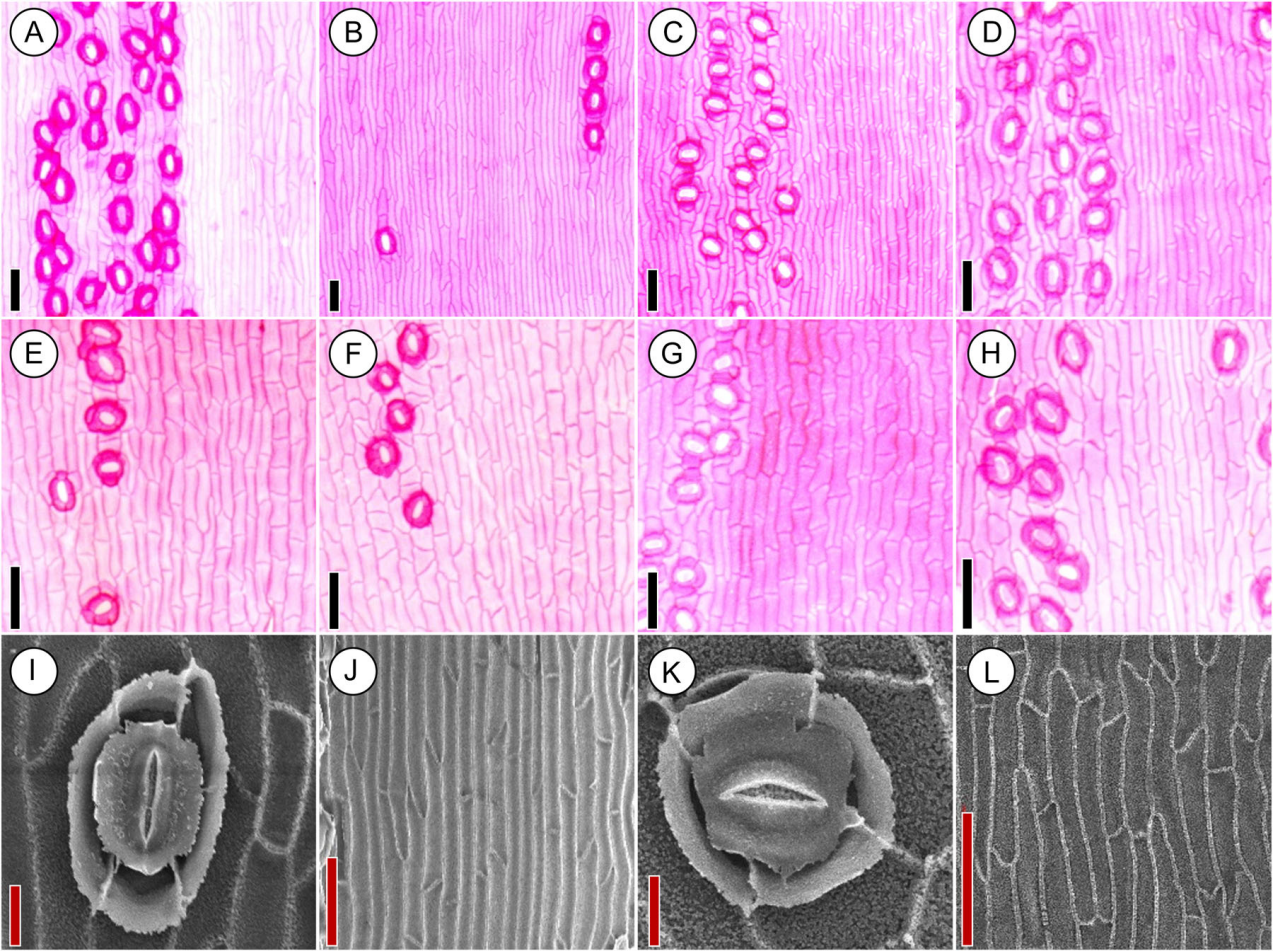
Epidermus of leaves at different heights on extant *Sequoia sempervirens*. (A–H) Leaves from 25 and 85 m heights on a wild tree at Prairie Creek Redwoods State Park (CA, USA). (A) Abaxial and (B) adaxial surface of linear leaf from 25 m height. (C) Abaxial and (D) adaxial surface of linear leaf from 85 m height. (E) Abaxial and (F) adaxial surface of scale leaf from 25 m height. (G) Abaxial and (H) adaxial surface of scale leaf from 85 m height. (I–L) Leaves from 5 m height of cultivated tree at the Tsukuba Botanical Garden. (I, J) SEM images of abaxial surface of linear leaf. (I) Stoma oriented parallel to the midrib; (J) elongated epidermal cells aligned along the midrib. (K, L) SEM images of abaxial surface of scale leaf. (K) Stoma oriented perpendicular to the midrib; (L) short epidermal cells. Scale bars: A–H, J, L = 100 µm; I, K = 20 µm.

**Figure 8:**
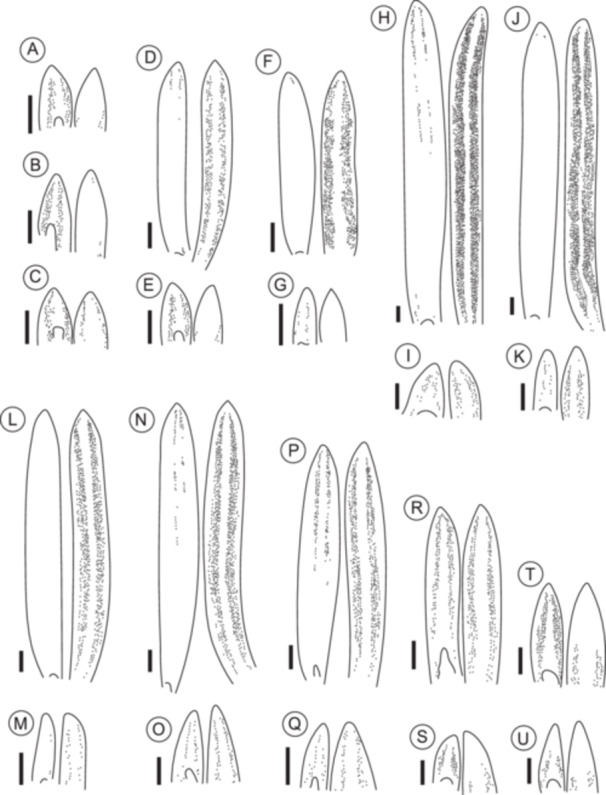
Diagrams of stomatal distribution patterns on linear and scale leaves on (A–G) *Sequoia primaria* and (H–U) extant *S. sempervirens*. In each panel, the adaxial side of leaf is on the left, the abaxial side of the same leaf is on the right. Except for A–C, vertically arranged pairs (e.g., D above E) were obtained from the middle (upper image) and basal (lower image) parts of the same branchlet. (A–E) Fossil leaves from the Yamabuki‐chō locality. Scale leaves in A–C were obtained from the central portions of separate shoots that did not bear linear leaves. (A) MTDO f‐1002‐25; (B) MTDO f‐1002‐32; (C) MTDO f‐1002‐30; (D) MTDO f‐1002‐14‐L1; (E) MTDO f‐1002‐14‐L3. (F, G) Fossil leaves from the Akadzu locality: (F) MTDO f‐1002‐68‐L1; (G) MTDO f‐1002‐68‐L3. (H–K) Leaves at 5 m height from a cultivated tree at the Tsukuba Botanical Garden. (H, I) Sun‐exposed leaves; (J, K) shaded leaves. (L–U) Sun‐exposed leaves from a wild tree at Prairie Creek Redwoods State Park at height of (L, M) 25 m, (N, O) 55 m, (P, Q) 70 m, (R, S) 85 m, (T, U) 100 m. Scale bar = 1 mm.

The epidermis of the linear and scale leaves of fossil samples was characterized by rectangular epidermal cells aligned parallel to the midrib (Figures 5A–D, F, H) and stomata surrounded by four to six subsidiary cells (Figures 5G, J). Variation in leaf length comparable to that observed in the fossils is also found in extant *Sequoia sempervirens* (Figure 6A–F). The epidermis of modern samples showed similar features, including rectangular epidermal cells aligned parallel to the midrib (Figure 7A–H, J, L) and stomata surrounded by four to six subsidiary cells (Figure 7I, K). Differences between linear and scale leaves included: (1) Linear leaves had a higher proportion of elongated epidermal cells than on scale leaves; (2) stomata on linear leaves were generally arranged parallel to the midrib, but irregularly arranged on scale leaves; (3) scale leaves often bore numerous stomata on the adaxial surface, whereas linear leaves had stomatal bands on the abaxial surface. However, gradual morphological transitions from scale to linear leaves were observed along shoots as leaf length increased, and the features described changed progressively in both fossil and modern samples (Figures 8, 9).

In the cone fossil specimen of *Protosequoia* described by Miki (1969), the terminal surface of the bract–scale complex exhibited a slight bulging. However, shallow transverse grooves comparable to those of *Sequoia* were observed on the terminal surface of the bract–scale complexes of those specimens and of specimens from the same locality (Figures 2A–C, 3E, 3F, 4H, 4I, 4K, 6G).

### Morphological variation in the leaves of extant *Sequoia sempervirens* with tree height

In samples from the wild trees of *S. sempervirens* in California exceeding 100 m in height, the length of the longest leaf on each shoot tended to decrease with increasing height (Figure 6C–F), as previously observed by Koch et al. (2004) and Van Pelt et al. (2016), with the longest leaves at the lowest height and on the shaded side of the tree (Figure 9). As tree height increased, the longest leaf on each shoot gradually had less basal twisting, was attached to the shoot at a more oblique angle, and eventually scale leaves were present (Figure 6C–F). Shoots in the upper canopy, at approximately 100 m in height, consisted solely of scale leaves that were tightly appressed to the shoot surface and exhibited short and thick annual increments (Figure 6F).

Shoots bearing only scale leaves were occasionally observed in the sun‐exposed canopy of cultivated individuals in Japan, at low elevations approximately 5 m above ground, similar to observations of the wild populations in California (Chin et al., 2022). These shoots occurred in the internodes between shoots with linear leaves, and their scale leaves were thinner and more widely spaced than those on shoots at higher positions on tall trees in California (Figure 6A, B).

The abaxial stomatal bands were clearly visible at all heights, whereas at 100 m, where all leaves were scale‐like, the bands were restricted to the lower portion of the leaf (Figure 8T, U). Adaxial stomatal bands on the scale leaves near the base of the shoot were also clearly visible at all heights (Figure 8I, K, M, O, Q, S–U). In contrast, on linear leaves below 55 m, adaxial stomatal bands were either absent (Figure 8L) or mainly appeared near the leaf apex and base (Figure 8H, J, N). At heights above 70 m, distinct adaxial stomatal bands began to appear from the center toward the apex of the leaf (Figure 8P), and by 85 m, they also extended to the leaf base (Figure 8R). At greater heights, stomatal orientation angles relative to the midrib tended to be wider and more irregular in all leaf types of similar size, accompanied by shorter epidermal cells on the abaxial and adaxial surfaces (Figure 9).

### Differences between fossil and modern specimens

Fossil linear leaves were generally shorter than those of modern *Sequoia sempervirens*. Even when leaves preserved as isolated specimens within the sediment were included, the maximum leaf length was typically 11 mm, and even in exceptionally elongated leaves, the length did not exceed 17 mm, whereas the length of linear leaves of modern *Sequoia* often exceeded 20 mm. The most common fossil linear leaves were approximately 8 mm long, comparable to the longest leaves on shoots at heights between 70 and 85 m in extant individuals. While scale leaves on shoots in the uppermost crown of *S. sempervirens* were completely appressed to the branches (Figure 6F), those of the fossil specimens frequently included leaves that spread outward from the branch, along with appressed forms (Figures 2E, 2F, 3B, 3E, 4A, 4B, 4E, 4G).

Stomata on the abaxial surfaces of scale leaves were less developed in fossil samples than in modern ones. Fossil scale leaves, including those on shoots with linear leaves, generally lacked stomata on their abaxial surfaces (Figure 8G) or had only a few stomata restricted to the apical or basal regions of the leaves (Figure 8A, B, E); the presence of two stomatal bands was rare (Figure 8C). In contrast, in extant specimens, stomata on the abaxial surfaces were typically distributed conspicuously from the leaf base to the apex (Figure 8I, K, M, O, Q). However, on leaves collected from crown positions above 85 m, stomata were restricted to the basal regions, resembling the condition observed in many fossil specimens (Figure 8S–U).

Stomata on the adaxial surface of fossil linear leaves were confined to the tip and/or base, typically arranged in one or two rows, each with fewer than 10 stomata (Figure 8D, F). A similar stomatal distribution was occasionally observed in the longest modern linear leaves at heights below 55 m (Figure 8H, J, N), whereas adaxial stomata were occasionally absent at lower positions (Figure 8L). Well‐developed adaxial stomata, as observed in modern linear leaves from heights above 70 m (Figure 8P, R), were not observed in fossil linear leaves.

A comparison of fossil and extant linear leaves of similar size revealed shorter epidermal cells and a lower frequency of irregularly oriented stomata on fossil samples (Figure 9). The epidermal morphology of fossil scale leaves closely resembled that of upper canopy leaves in extant tall trees, regardless of whether they occurred on shoots dominated by linear leaves or on shoots bearing only scale leaves. The epidermal cell length and stomatal orientation on both surfaces were comparable to those of leaves from higher positions on extant trees, where shorter cell lengths and irregularly oriented stomata were typical (Figure 9).

The seed cones of both fossil and extant species were similar in size and shape. Their bract–scale complexes shared a rhombic‐shaped terminal surface with a shallow transverse groove at the center (Figures 2A–C, 3E, 3F, 4H, 4I, 4K, 6G). Numerous stomata were observed on the adaxial side of this terminal surface in both fossil and extant species (Figure 10). In cross section, the fossil bract–scale complexes had less developed vascular bundles, but their overall arrangement was generally rhomboid, similar to that of the extant specimens (Figure 11). Fossil seed cones were attached to shoots that predominantly bore scale leaves (Figures 2D, E and 3F).

### Systematics of fossil *Sequoia*

***Family***—Cupressaceae Gray (1822)

***Subfamily***—Sequoioideae (Luerss.) Quinn (2000)

***Genus***—*Sequoia* Endl. (1847)

***Species***—*Sequoia primaria* (Miki) Ikeda et Momohara comb. nov.

***Basionym***—*Sequoiadendron primarium* Miki (1965), p. 3, Figure 1A, p. 4, Plate 1A–E.

***Synonyms***—*Sequoia sempervirens* (D. Don) Endl.: Miki (1941), p. 258, Figure 7G, IA–E, p. 305, Plate 5E, F. *Sequoia sempervirens* (D. Don) Endl.: Miki (1948), p. 141, Plate 4, Figure Da. *Sequoiadendron* cf. *chaneyi* Axelrod: Miki (1963), p. 93, Plate A. *Sequoia couttisie* Heer: Miki (1965), p. 3, Text Figure 1C; p. 5, Plate 2A–C. *Protosequoia primaria* (Miki): Miki (1969), p. 728, Figure 1; p. 729, Figure 2A.

***Syntypes***—The original materials illustrated as Plate 1 Aa, Ab, B, Ca, Cb, Cc, Cd, D, and E by Miki (1965) are here designated as syntypes. Of these, the following specimens were located and confirmed in the OSA Collection: OSA F11829 (Miki, 1965: Plate 1Cb), illustrated here in Figure 2A; OSA F2296 (Miki, 1965: Plate 1Aa), here in Figure 3A; OSA F2651 (Miki, 1965: Plate 1Ca); OSA F2194 (Miki, 1965: Plate 1 Cd), here in Figure 3F; from and OSA F2657 (Miki, 1965: Plate 1E), here in Figure 3I and J. The remaining specimens illustrated by Miki (1965) as Plate 1Ab, B, Cc, and D could not be located and are therefore treated here as untraced original material.

***Lectotype***—OSA F11829 (Miki, 1965: Plate 1Cb) shown here in Figure 2A.

***Epitype***—OSA F2070 (Miki, 1969: Figure 1 Aa), shown in Figure 2D, collected from Akadzu, Seto City, Aichi Prefecture, Japan.

***Type locality***—Ōbora, border between Tajimi and Toki Cities, Gifu Prefecture, Japan.

***Type strata***—Upper Miocene Tokiguchi Porcelain Clay Formation, Seto Group.

Other macrofossil specimens examined and their figure numbers: OSA F2069 (Figure 3E); OSA F2630 (Miki, 1941: left side of Plate 5 F; here: Figure 3D); OSA F2635 (Miki, 1941: right side, Plate 5 F); OSA F2641 (Miki, 1969: Figure 1 Ad); OSA F2646, originally Figure 1 Ac of Miki, 1969; OSA F11527 (Figure 3C); OSA F11833 (Miki, 1969: Figure 2 Aa); OSA F12132 (Figure 3B); MTDO f‐1002‐1 (Figure 4A); MTDO f‐1002‐2 (Figure 4B); MTDO f‐1002‐3 (Figure 4C); MTDO f‐1002‐4 (Figure 4D); MTDO f‐1002‐5 (Figure 4E); MTDO f‐1002‐6 (Figure 4H); MTDO f‐1002‐7 (Figure 4I); MTDO f‐1002‐8 (Figure 4J); MTDO f‐1002‐9 (Figure 4K); MTDO f‐1002‐10 (Figure 4L); MTDO f‐1002‐11 (Figure 4M); MTDO f‐1002‐12 (Figure 4N).

### Emended diagnosis

Shoots dimorphic and often branched, comprising scale‐leaved shoots predominantly bearing scale leaves with obtuse to acute apices and linear‐leaved shoots mainly bearing linear leaves, with an abrupt transition between scale and linear leaves along a single shoot. Linear leaves with two distinctly wide stomatal bands on the abaxial surface and small stomatal zones near leaf apex and/or base on the adaxial surface. Scale leaves with narrow stomatal zones often restricted to the leaf base on the abaxial surface and two stomatal bands on the adaxial surface. Seed cones spherical to ellipsoidal, consisting of helically arranged bract–scale complexes; distal surface of each bract–scale complex with a shallow horizontal groove, rarely slightly convex; numerous stomata on the adaxial side. Seeds irregularly ovate with broad lateral wings. Male strobili spherical to ovoid.

### Description

Shoots with helically arranged leaves are dimorphic and often branched, comprising scale‐leaved shoots predominantly bearing scale leaves (Figures 2E, 3A, 3E, 3I, 3J, 4A, 4D) and linear‐leaved shoots mainly bearing linear–lanceolate leaves that appear two‐ranked, with scale leaves present at the base and near the shoot tip (Figures 3B–D, 3G, 4C, 4E). Within a single shoot, the leaf form abruptly changed between the scale and linear forms, with leaves morphologically intermediate between the two forms (Figures 3B–D, G and 4B, C, E). Shoots bearing linear leaves are 16–35(–55) mm long, with a shoot axis 0.5–1.0 mm wide. Shoots bearing only scale leaves are 15–70(–170) mm long, with a shoot axis 0.8–1.6 mm wide. The base of the shoot occasionally is 1.4–6.0 mm wide.

Linear leaves are linear or linear‐lanceolate, 2–11 (rarely to 17) mm long and 0.8–1.5 mm wide, with a length to width ratio (L/R) of 5.2–11.3. The apex is acute; the base is slightly narrower slightly and significantly twisted at an angle of 35–60°(–80°) before becoming adnate to the shoot axis (Figure 4F). Scale leaves in adaxial view are triangular, with an acute to rounded apex, 0.8–2.0 mm long and 0.7–0.9 mm wide, with L/R of 1.0–1.9, broadly adnate to the shoot axis at the base. Leaves of the same length as scale leaves may occasionally become subulate; such leaves are often intermixed among shoots on which scale leaves predominate (Figure 4G).

Leaves are amphistomatic (Figure 8A–F), though scale leaves are occasionally epistomatic (Figure 8G). Stomatal bands on linear leaves occur on both sides of the midrib, each consisting of 2–5 rows of stomata extending from the base to near the apex on the abaxial surface (Figure 8D, F). On the adaxial surface, stomata occur in one to three rows near the tip and/or base (Figure 8D, F). Stomatal bands on scale leaves occur in 2–5 rows along the midrib on the adaxial surface, and in 0–3 rows along the edge of the lower part on the abaxial surface (Figure 8A, B, C, E, G).

Stomata in linear leaves are predominantly arranged parallel to the midrib at an angle of 0–14° (–45°) (Figure 5A, E, G). In scale leaves, stomata are predominantly oriented diagonally on the adaxial surface, at an angle of 11–55° (2–84°) to the midrib (Figure 5D, I), and arranged irregularly on the abaxial surface, at an angle of (5°–) 30–90° to the midrib (Figure 5C, H, J). Each stoma is surrounded by four or five, occasionally six, subsidiary cells (Figure 5G, J). Stomata, including subsidiary cells, are oval, in linear leaves, 40–71 μm long and 34–52 μm wide, with L/R of 1.1–1.8; in scale leaves, 46–71 μm long and 36–63 μm wide, with L/R of 0.8–1.9.

In the nonstomatal areas, epidermal cells of linear leaves are narrowly rectangular, occasionally parallelogram‐ or trapezoid‐shaped, with their long sides parallel to the midrib (Figure 5A, B, F); on abaxial surface, 53–174 (–322) μm long and 7–27 μm wide, with L/R of 2.6–13.9 (–23.2) on adaxial surface, 55–153 (–216) μm long and 6–21 μm wide, with L/R of 3.1–10.7 (–17.4). On scale leaves, epidermal cells are similarly parallelogram‐ or trapezoid‐shaped with their long sides parallel to the midrib (Figure 5C, D), but cells are shorter and thicker (Figure 5I); on abaxial surface, 21–92(–173) μm long and 9–35 μm wide, with L/R of 1.0–6.2(–10.9) and on adaxial surface, 22–103 (–192) μm long and 6–25 μm wide, L/R of 1.2–8.7(–16.9). In the stomatal area, epidermal cells of linear leaves are elongated polygonal, ranging from triangular to hexagonal, with slightly curved anticlinal walls (Figure 5A, E), 24–95(–156) μm long and 8–24 μm wide, with L/R of 2.3–10.8. Epidermal cells of scale leaves are also polygonal in shape with slightly curved anticlinal walls (Figure 5C, D, H), 33–103(–148) μm long and 11–25 μm wide, with L/R of 1.5–7.1.

Seed cones are spherical to ellipsoidal (Figures 2A, 2B, 3E, 3F, 4H, 4I), 12–23(–28) mm long and 10–16 mm wide, with L/R of 1.0–1.5; and occur solitary at the tip of shoots composed of scale leaves (Figures 2D, 2E, 3E). The bracts at the base of the seed cone have an acute to rounded apex (Figure 2D). The cone consists of 18–26 bract–scale complexes arranged helically. In upper view, the bract–scale complex is fan‐shaped (Figures 2C, 3F, 4J, 4K), 5.1–6.2 mm from the peduncle base to the terminal surface and 6.1–9.6 mm wide at the terminal surface. The terminal surface is rhombic with horizontal transverse grooves (Figures 2C, 3F, 4H, 4I, 4K), although it becomes slightly convex in immature cones (Figures 2E, 3E) and bears numerous stomata radiating from the center to the adaxial side (Figure 10A, B). In transverse sections, the vascular bundles of the bract–scale complex are typically arranged in a rhomboid or reniform pattern and divided into 20–30 strands near the distal end, with 1–4 resin canals frequently present on the abaxial side (Figure 11A, C). On the adaxial surface, 2–5 seeds are arranged in an inverted single row. The seeds are flattened and irregular oval (Figures 4L, M), 4.1–6.0 mm long and 3.1–5.1 mm wide, and have broad wings on both sides.

Immature male strobili are spherical or ovoid (Figures 3I, 3J, 4N) and are attached singly to the branchlet, 2.3–4.1 mm long, 2.1–4.0 mm wide, with L/R of 0.8–1.4. Distal part of the mature male strobilus is composed of approximately 12 spirally arranged microsporophylls (Figure 3H).

## DISCUSSION

### Taxonomic position of *Sequoia primaria*

*Sequoia primaria* from the Seto and Tokiguchi Porcelain Clay Formations was initially reported by Miki (1963) as the fossil‐species *Sequoiadendron* cf. chaneyi Axelrod and later described as *Sequoiadendron primarium* Miki (Miki, 1965). Dr. Miki classified this fossil as *Sequoiadendron*, rather than *Sequoia*, based on several distinguishing characteristics: shoots bearing only scale leaves with stomata arranged diagonally to the midrib, a convex and thick bract–scale complex, prominent stomata on the distal surface of the bract–scale complex, shorter scale leaves (referred to as “bracts” by Miki, 1969) at the base of the cone, and the absence of seed wings (Miki, 1963, 1965, 1969). Subsequently, Miki (1969) reassigned the species to a new genus, *Protosequoia*, distinguishing it from *Sequoiadendron* based on characteristics such as smaller cones, fewer rows of convex and thick bract–scale complexes, and the presence of abscission layers in the shoots. Fossil *Sequoia* from the same formations was initially identified as the extant species *Sequoia sempervirens* (Miki, 1941) but later reassigned to *Sequoia couttsiae* Heer (Miki, 1965). This reassignment was based on the absence of scale leaves at the base of the cone and the presence of an elongated shoot portion bearing scale leaves as shown in Figure 3D. In that study, Miki (1965) also classified other Japanese *Sequoia* fossils as *S. couttsiae*. However, *S. couttsiae* has since been reclassified into the genus *Quasisequoia* (Kunzmann, 1999).

We propose that the dimorphic shoots previously classified as separate genera, *Sequoia* and *Protosequoia*, originated from the same species. This interpretation is supported by multiple lines of evidence: (1) fossil shoots preserved in connection, exhibiting morphological features attributed to both genera (Figures 3B–D, G and 4B, C, E); (2) the presence of small linear leaves in fossil specimens used by Miki (1969) to define *Protosequoia* (Figure 2F); and (3) a continual range of variation in epidermal cell length and stomatal angle relative to leaf length within a single shoot (Figure 9). Detailed comparisons of reproductive organs further support this taxonomic revision. Miki (1969) noted that unlike *Protosequoia*, which bears numerous stomata, the distal surface of the bract–scale complex in *Sequoia* has almost no stomata, which he regarded as diagnostic for the genus. However, cones of both *Protosequoia* and modern *S. sempervirens* have numerous stomata concentrated on the adaxial side on the distal surface of the bract–scale complexes (Figure 10). Therefore, this trait is not a reliable taxonomic distinction. The bract–scale complexes of *Protosequoia* are generally slightly more convex on the terminal surface than those of *Sequoia*. However, a horizontal groove across the terminal surface, a characteristic feature of *Sequoia*, is also observed in *Protosequoia* specimens (Figures 2A–C, 3E, 3F, 4H–K). For scale leaves at the base of the seed cone, Miki (1969) noted that those of *Sequoia* are more aristate than those of *Protosequoia*; however, considerable individual variation exists, and the leaves are often not elongated (Figure 6G). Miki (1969) stated that the seeds of *Protosequoia* lack prominent wings; however, due to variation in the thickness of the seed margin, the boundary between the seed body and wing is difficult to discern from fossils, as shown in Figure 4M. Therefore, the presence or absence of the wing is also not a reliable taxonomic distinction.

We designated the cone shown in Plate 1Cb of Miki (1965) as the lectotype of *S. primaria* (OSA F11829; Figure 2A). However, this specimen alone does not provide sufficient information regarding the leaf morphology diagnostic for this fossil‐species. Therefore, we designated a well‐preserved specimen from Akadzu, shown in Figure 1A of Miki (1969), as the epitype of *S. primaria*. This specimen was selected because it exhibits, in organic connection, the three diagnostic morphological features considered critical for applying the name: a seed cone, a densely branched scale‐leaved shoot, and the presence of a few small linear leaves (OSA F2070; Figure 2E). This combination of diagnostic features is not fully represented in any single specimen among the original syntype material of Miki (1965).

### Comparison between extant and fossil Sequoioideae species

Most features of *Sequoia primaria* closely resemble those of *Sequoia*. Among the closely related genera within Sequoioideae, *Sequoiadendron*, *Quasisequoia*, and *Austrosequoia* bear small scale leaves on the shoots but lack linear leaves characteristic of *Sequoia*. In these genera, the leaf morphology tends to shift toward a subulate form as leaf length increases; however, in *S. primaria*, leaves exceeding 2 mm long are consistently flat and linear. Additionally, the seed cones of *Sequoia* typically comprise fewer than 30 bract–scale complexes, whereas *Sequoiadendron giganteum* (Lindl.) J. Buchholz bears 28–45 very thick bract–scale complexes (Kunzmann, 1999; Farjon, 2005). The terminal surfaces of the bract–scale complexes in *Quasisequoia* are predominantly hexagonal and distinctly convex (Srinivasan and Friis, 1989; Kunzmann, 1999). In contrast, the terminal surfaces of the bract–scale complexes in *Sequoia* are rhombic and feature a distinct transverse groove. Fossil records of *Austrosequoia* are restricted to the Cretaceous of Australia and surrounding regions, with no reliable records documented from the Cenozoic (Peters and Christophel, 1978; Mays et al., 2018). *Metasequoia*, likewise belonging to Sequoioideae, differs from *Sequoia* in bearing planar, exclusively lanceolate to linear leaves arranged oppositely. Despite the general similarity of their seed cones, *Metasequoia* is readily distinguished by its decussately arranged bract–scale complexes (Kunzmann, 1999; Farjon, 2005).

Fossil *Sequoia* species from the Cenozoic in Japan and China have been assigned to *S. affinis* Lesquereux (Tanai, 1961), *S. hondoensis* Yasui (Yasui, 1917), *S. langsdorfii* (Brongniart) Heer (Ishida, 1970; Uemura, 1988), *S. chinensis* (Endo) Wang et Li (WGCPC, 1978; Ma et al., 2005), and *S. maguanensis* Zhang et Zhou (Zhang et al., 2015), in addition to *S. couttsiae*, to which Miki (1965) referred most of the fossil remains from Japan. Exceptions include fossil *Sequoia* species such as *S. japonica* Endo and *S. onukii* Endo, which were later reassigned to *Metasequoia occidentalis* (Newberry) Chaney (Liu et al., 1999). *Sequoia hondoensis*, described from wood fossils found in coal fields in Aichi Prefecture, Japan, exhibits anatomical features resembling those of *Sequoia* (Yasui, 1917). However, it is currently not feasible to confidently assign wood and shoot fossils to the same species, and wood fossils are often classified under different names, such as *Sequoioxylon*. Based on these considerations, *S. primaria* is described separately from *S. hondoensis*. *Sequoia langsdorfii*, initially described as *Taxites* by Brongniart (1828) and later reassigned to *Sequoia* by Heer (1855), is now considered synonymous with *Sequoia abietina* (Brongniart) Knobloch (1964) (Kvaček, 1976). *Sequoia abietina* was originally described as *Phyllites* by Cuvier and Brongniart (1822). Thus, fossil *Sequoia* species referable to *S. primaria*, identified based on shoots and cones from Cenozoic strata, currently include *S. affinis*, *S. abietina*, *S. chinensis*, and *S. maguanensis*.

*Sequoia primaria* can be distinguished from these fossil species by its densely branched shoots bearing well‐developed scale leaves, a distinctive morphological feature among fossil *Sequoia* species (Table 1). Similarly developed shoots with scale leaves have been reported in *S. affinis* (e.g., Chaney, 1951: Plate 3, Figure 3); however, they are less branched than *S. primaria*. Additionally, the seed cones of *S. primaria* are larger, whereas those of *S. affinis* are approximately 10 mm in diameter (Chaney, 1951; MacGinitie, 1953). *Sequoia abietina* is a heterophyllous conifer bearing predominantly linear leaves, but also short scale, acicular, or subulate leaves, with foliar variability closely resembling that of extant *S. sempervirens* and fossil *S. primaria*. However, *Sequoia abietina* can be distinguished from *S. primaria* by its significantly larger linear leaves, which reach 31 mm in length and 2.8 mm in width (Grímsson et al., 2007; Worobiec and Worobiec, 2019). In addition, shoots predominantly composed of spreading subulate cryptomerioid leaves have been reported in *S. abietina* (Worobiec and Worobiec, 2019). In contrast, *Sequoia primaria* lacks shoots composed solely of such cryptomerioid leaves; instead, small subulate leaves are occasionally and irregularly intermixed with appressed scale leaves (Figure 4G), frequently with a sudden transition between scale leaves and linear leaves along the shoot. Considering the morphology of its linear leaves and its geographic distribution, *S. primaria* is likely most closely related to *S. chinensis* and *S. maguanensis*, both of which have been reported from China. However, taxonomic comparisons with these fossil species are challenging because they were primarily described based on shoots bearing linear leaves. *Sequoia chinensis* is characterized by hypostomatic leaves, whereas *S. primaria* and *S. maguanensis* have amphistomatic leaves (Ma et al., 2005; Zhang et al., 2015). However, owing to considerable variation in stomatal distribution on the adaxial surface of linear leaves even within extant *S. sempervirens* (Figure 8H, J, L, N, P, R), the taxonomic reliability of this character in fossil species remains uncertain. *Sequoia maguanensis* produces elliptical male strobili (Zhang et al., 2015), whereas *S. primaria* bears globose to ovoid male strobili, providing an additional distinguishing feature between the two species.

**Table 1:** Morphological characteristics of *Sequoia* species and related genera. Leaf epidermal cells on the nonstomatal zone of both surfaces were measured. References: (a) this study; (b) Kunzmann (1999); (c) Farjon (2005); (d) Zhang et al. (2015); (e) Kvaček (1976); (f) Teodoridis (2003); (g) Grímsson et al. (2007); (h) Worobiec et al. (2008); (i) Barrón and Postigo‐Mijarra (2011); (j) Worobiec and Worobiec (2019); (k) Chaney (1951); (l) MacGinitie (1953); (m) Meyer (2003); (n) WGCPC (1978); (o) Ma et al. (2005); (p) Srinivasan and Friis (1989); (q) Peters and Christophel (1978); (r) Mays et al. (2018).

Species or genus	Age	Distribution	Leaf shape	Scale–subulate leaf					Linear leaf				
				L × W (mm)	Apex	Shoot	Stomata L × W (μm)	Epidermal cell L × W (μm)	L × W (mm)	Apex	Shoot length (mm)	Stomata L × W (μm)	Epidermal cell L × W (μm)
***Sequoia primaria***	Late Miocene	Central Japan	Scale‐subulate‐linear	0.8–2.0 × 0.7–0.9	Obtuse–Acute	Highly developed (densely branched)	46–71 × 36–63	21–192 × 6–35	2–11(–17) × 0.8–1.5	Acute–acuminate	16–35 (–55)	40–71 × 34–52	53–322 × 6–27
***S. sempervirens***	Extant	North America	Scale‐subulate‐linear	1.2–10.0 × 0.9–1.5	Acute	Developed (sparsely branched)	55–78 40–70	26–267 × 9–25	6–31 × 1–2.5	Acute–acuminate	27–114	60–88 × 40–60	54–541 × 6–24
***S. abietina***	Eocen–Miocene	Europe	Scale‐Subulate‐Linear	Up to 6 × 1.0–1.5	Acute	Developed? (sparsely branched)	41–66 × 24.0–41.7	Up to 73.5 × 12.3–24.5	7–31 × 0.5–2.8	Acute	36.2–75	39–71.3 × 24.0–46.7	60–250 × 9.8–24.5
***S. affinis***	Oligocene–Pliocene	North America	Scale‐subulate‐linear	—	Acute	Developed (sparsely branched)	—	—	5–18 × —	Obtuse–acute	—	—	—
***S. chinensis***	Eocene	Northeast China	Scale‐linear	—	—	Uncertain	—	—	4–14 × 0.6–1.5	Mucronate	36–80	60–72 × 36–54	33–120 × 15–33
***S. maguanensis***	Late Miocene	South China	Scale‐linear	1.2–2.2 × 0.6–1.0	Obtuse?–Acute	Uncertain	—	—	6.6–9.2 × 0.9–1.1	Acute	32–62	63.4–82.4 × 37.9–59.3	60.5–148.7 × 12.1–21.1
***Sequoiadendron giganteum***	Extant	North America	Scale‐subulate	1–20 × 1–5	Acute	This only (Densely branched)	56–68 × 32–44	20–210 × 8–44	—	—	—	—	—
***Quasisequoia***	Cretaceous–Miocene	Europe	Scale‐subulate	0.7–7 × 0.4–2	Acute	This only (Sparsely branched?)	36–125 × 25–112.5	15–125 × 15–37.5	—	—	—	—	—
***Austrosequoia***	Cretaceous–Oligocene?	Australia, New Zealand	Scale–Subulate	2.8–9.2 × 1.2–2.6	Acute	This only (Sparsely branched?)	44–94 × 32–68	13–56 in diameter	—	—	—	—	—

Species or genus	Seed cone			Bract–scale complex			Seed			Male strobilus		
	shape	Length × width (mm)	No. bract–scale complexes	Terminal surface shape	Length × width (mm)	No. rows of seeds	Shape	Diameter (mm)	Wing	Shape	Diameter (mm)	Reference
***Sequoia primaria***	Ellipsoidal or globose	12–23(–28) × 10–16	18–26	Rhomboidal and slightly convex or markedly concave	5.1–6.2 × 6.1–9.6	1	Irregular oval	4.1–6.0 × 3.1–5.1	Wide	Spherical or ovoid	2.3–4.1 × 2.1–4.0	a
***S. sempervirens***	Ellipsoidal	15–30 × 13–18	18–29	Rhomboidal and markedly concave	4–5 × 6–10	1	Irregular oval	4.5–6 × 3–4	Wide	Spherical	5 × 4	a–d
***S. abietina***	Ovoid or globose	2.9–21.6 × 2.6–15.0	10–12	Rhomboidal and slightly convex or markedly concave	0.6–1.5 × 0.9–9.6	1	Ovate or triangular	2.5–3.2 × 1.6–1.9	Wide	—	—	e–j
***S. affinis***	Ovoid or globose	About 10 × 10	9–12	Rhomboidal and markedly concave	? × 3–5	—	—	—	—	Spherical or ovoid	3–5	k–m
***S. chinensis***	Globose	About 15 × 14	About 20	Rhomboidal and markedly concave	? × 8	—	—	—	—	—	—	n, o
***S. maguanensis***	—	—	—	—	—	—	—	—	—	Oblong or ellipsoidal	3 **×** 2	d
***Sequoiadendron giganteum***	Ellipsoidal or ovoid	30–80 × 25–50	28–45	Rhomboidal and strongly concave	6–12 × 7–30	2	Irregular oval	4–7 × 3–5	Wide	Subglobose	4–6 × 3–5	b, c
***Quasisequoia***	Ellipsoidal	8–25 × 8–20	20–51	Rhomboidal–Hexagonal and markedly convex	2.2–7 × 2.5–8	1	Ellipsoidal?	1.6–6.0 × 1–4.5	Inconspicuous	—	—	b, p
***Austrosequoia***	Ellipsoidal or globose	9–19.9 × 6–22.6	19–49	Rhomboidal and markedly concave	1.5–2.5 × 3.5–4.5	1	Ellipsoidal or ovate	1.3–1.6 × 0.4–0.7	Inconspicuous	—	—	q, r

*Note*: A dash (—) indicates that information was not provided or applicable.

For Cretaceous fossils of Sequoioideae, anatomical details of the bract–scale complexes, including the size and arrangement of vascular bundles and resin canals and specific features such as trichomes, have been extensively compared (Ohsawa et al., 1992; Ohsawa, 1994; Rothwell and Ohana, 2016; Sokolova et al., 2017). In contrast, comparable detailed analyses are rare for fossil *Sequoia*, making interspecific comparisons based on these characteristics challenging.

### Ecological implications of leaf forms in *Sequoia primaria*

In *Sequoia sempervirens* individuals exceeding approximately 90 m in height, the maximum leaf size on a shoot decreases with increasing shoot height along the tree, and ultimately, entire shoots at the top of the crown are covered exclusively with scale leaves (Koch et al., 2004). This reduction in leaf size is believed to result from a decline in water potential from the base to the apex of the tree, which reduces turgor pressure and photosynthetic capacity necessary for leaf development (Koch et al., 2004; Ishii et al., 2008). Although in *S. sempervirens*, the size of linear leaves composing a shoot tends to be uniform at a given height, *S. primaria* often shows a transition within the same branch, from shoots bearing only linear leaves to those composed entirely of scale leaves. Fossil linear leaves, characterized by extremely low stomatal density on the adaxial surface, resemble leaves from lower positions in extant *Sequoia*, where water potential is relatively high. In contrast, scale leaves, mainly appressed to the stem and lacking stomata on the outer abaxial surface or possessing stomata only at the leaf base, resemble those in the upper crown of extant tall trees, where water potential is minimal. The observed transition in leaf form and stomatal distribution suggests that, in *S. primaria*, a pattern comparable to the height‐related leaf form variation in *S. sempervirens* occurs along a single branch. Thus, morphological variation in leaf type may reflect changes in water stress during shoot development rather than differences in tree height. Supporting this interpretation, similar changes in leaf size on a single shoot were observed in an experiment in which a shoot bearing only scale leaves from the upper crown, when planted in moist soil, subsequently developed linear leaves (Koch et al., 2004). This evidence further indicates a degree of plasticity in leaf size for single shoots of *Sequoia* depending on hydraulic conditions.

*Sequoia primaria* co‐occurs with plant fossils indicative of riparian wetland environments, such as *Eoeuryale*, *Trapa*, *Carex*, and *Schoenoplectiella* and has therefore been previously assumed to have inhabited humid environments with an oceanic climate and minimal amplitude in the annual temperature (Miki, 1963, 1969; Momohara and Saito, 2001). However, a micromorphological analysis of paleosols from the Seto and Tokiguchi Porcelain Clay Formations, where *S. primaria* also occurs, by Hatano and Yoshida (2017, 2018) revealed evidence of a seasonal dry period. These formations, deposited between 11 and 9 Ma (Hatano and Yoshida, 2017), correspond to the period when the East Asian monsoon began to develop around 11 Ma and peaked between 8 and 9 Ma (Zheng et al., 2004).

In coastal regions of California and Oregon, where *S. sempervirens* is currently found, winter rainfall and frequent summer fog creates a humid and relatively mild climate with minimal annual temperature variation (Dawson, 1998; Sawyer et al., 2000). In such low‐water‐stress habitats, variation in leaf size along tree height is primarily driven by height‐related gradients in water availability. In contrast, seasonal droughts during the earliest Late Miocene in East Asia may have caused variation in leaf size within individual shoots of *S. primaria*, reflecting within‐shoot responses to seasonal fluctuations in water availability rather than differences associated with tree height.

The predominance of scale leaves that we documented here provides new insight into the evolutionary history of *Sequoia*. At the same time, external morphology alone is unlikely to resolve the specific ecological drivers underlying the dominance of this leaf form. In extant *Sequoia*, constraints associated with extreme tree height have been interpreted largely based on leaf anatomical and functional evidence. Extending comparable anatomical, functional, and height‐related frameworks to fossil material may therefore enable a more refined reconstruction of the ecology of fossil *Sequoia* and the selective pressures that shaped the foliar architecture of *S. sempervirens*.

## AUTHOR CONTRIBUTIONS

Conceptualization: S.I., A.M.; methodology: S.I., A.M.; investigation: S.I., A.M.; formal analysis: S.I.; funding acquisition: S.I., A.M.; visualization: S.I.; writing original draft: S.I.; review and editing: S.I., A.M.; supervision: A.M.

## CONFLICT OF INTEREST STATEMENT

The authors declare they have no conflict of interest.

## Data Availability

All fossil specimens examined in this study are housed at Graduate School of Horticulture, Chiba University and the Osaka City Museum of Natural History. Specimen numbers for the figured are provided in the Results section. The complete fossil specimen list (*N* = 780) and quantitative data for Figure 9 are archived in Dryad at https://doi.org/10.5061/dryad.sxksn03hf. Epidermal cell length and stomatal angle on abaxial and adaxial sides relative to leaf length on same branchlet in fossil *Sequoia primaria* from different localities and on leaves from different heights on cultivated or wild trees of extant *S. sempervirens*. In each graph, data points from left to right represent scale leaves at the base, intermediate leaves in the middle, and linear leaves in the middle of a branchlet. Each point represents the mean of leaves from three branchlets in fossil specimens and the mean of leaves from five branchlets from living trees. Vertical bars indicate standard deviations. In *S. sempervirens*, 5 m denotes leaves from 5 m height from a cultivated tree at the Tsukuba Botanical Garden; 25 m, 55 m, 70 m, and 85 m denote leaves collected from a wild tree at Prairie Creek Redwoods State Park. Crown positions: sun, sun‐exposed; shade, shaded. Leaf surfaces: Ab, abaxial; Ad, adaxial. Light micrographs of safranin‐stained epidermal preparations showing stomatal distribution on terminal surface of bract–scale complex. (A, B) Fossil *Sequoia primaria* comb. nov. from the Yamabuki‐chō locality (MTDO f‐1002‐45). (A) Right half of terminal surface; arrow indicates the center of the transverse groove that separates the adaxial (upper) and abaxial (lower) sides; (B) adaxial side with numerous stomata. (C, D) Living *S. sempervirens* from the Tsukuba Botanical Garden. (C) Entire terminal surface, showing the prominent transverse groove separating the adaxial (upper) and abaxial (lower) sides; (D) adaxial side with numerous stomata. Scale bars: A, C = 1 mm; B, D = 100 µm. Serial transverse sections of bract–scale complexes, arranged from the terminal to basal position, with adaxial side at the top of each section. (A) From fossil *Sequoia primaria* comb. nov. from the Yamabuki‐chō locality (MTDO f‐1002‐44). (B) Line drawings of sections in (A). (C) From living tree of *S. sempervirens* from the Tsukuba Botanical Garden. (D) Line drawings of sections in (C). R (red), resin canals; V (black), vascular bundles. Scale bar = 1 mm.
